# Tools to reverse-engineer multicellular systems: case studies using the fruit fly

**DOI:** 10.1186/s13036-019-0161-8

**Published:** 2019-04-23

**Authors:** Qinfeng Wu, Nilay Kumar, Vijay Velagala, Jeremiah J. Zartman

**Affiliations:** Department of Chemical and Biomolecular Engineering, University of Notre Dame, Notre Dame, IN 46556 USA

**Keywords:** Microfluidics, Image processing, Machine learning, Deep learning, Imaging

## Abstract

Reverse-engineering how complex multicellular systems develop and function is a grand challenge for systems bioengineers. This challenge has motivated the creation of a suite of bioengineering tools to develop increasingly quantitative descriptions of multicellular systems. Here, we survey a selection of these tools including microfluidic devices, imaging and computer vision techniques. We provide a selected overview of the emerging cross-talk between engineering methods and quantitative investigations within developmental biology. In particular, the review highlights selected recent examples from the *Drosophila* system, an excellent platform for understanding the interplay between genetics and biophysics. In sum, the integrative approaches that combine multiple advances in these fields are increasingly necessary to enable a deeper understanding of how to analyze both natural and synthetic multicellular systems.

## Background

**Fig. 1:**
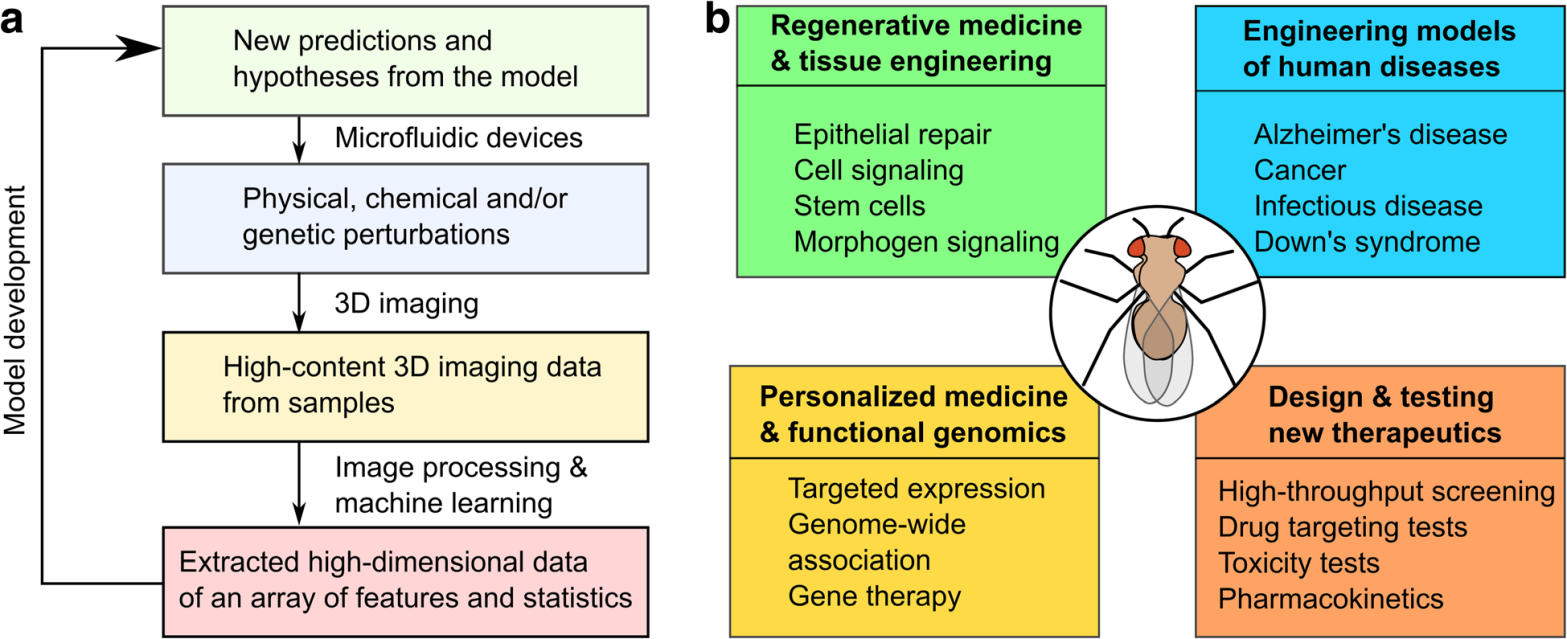
Workflow for reverse-engineering multicellular systems and the broad applicability of *Drosophila* as an integrative test case. **a** A prototypical, iterative flow for systems analysis of multicellular systems consists of using microfluidic devices to precisely manipulate tissue samples, advanced imaging technologies to generate high-content data, image processing pipeline such as machine learning for data extraction and computational modeling for hypothesis revision and regeneration. **b** *Drosophila* is an excellent model organism for investigating a broad range of grand challenges in systems biology and bioengineering. For regenerative medicines, *Drosophila* helps identify physiological processes involved in wound closure. *Drosophila* also serves as models for many human diseases, such as Alzheimer’s disease and cancer. For personalized medicine and functional genomics, the effects of alternative gene mutations can be mapped to phenotype. *Drosophila* also serves as a high-throughput platform for drug screening that is physiologically relevant to human

Answers to many human health challenges require an integrated systems-level understanding of the body [1]. Biocomplexity, the emergence of properties that are more than the sum of individual constituents, leads to profound implications on how to solve problems in regenerative medicine, cancer therapy, and personalized medicine [2]. This complexity spans multiple spatial scales from molecules, such as proteins and DNA, to cells, tissues, organs and organ systems. It requires a systems-level analysis to understand this complexity [3]. The general paradigm of systems research adopts an iterative approach, which usually involves transitioning from experiments to model formulation then to revision of original hypotheses (Fig. 1a) [4].

Genetic model systems, such as the worm—*C. elegans*, the zebrafish or the fruit fly—*Drosophila melanogaster*, serve as proof-of-principle platforms for developing tools to analyze multicellular systems or to test new techniques in forward-engineering living systems [5]. In particular, *Drosophila* enables genetic studies of how genes are regulated to control morphogenesis [6–8] and physiology [9]. It is an excellent system for studies that are at the crossroad of biophysics, information processing, and molecular and developmental biology. The fruit fly system provides many advantages, including cheap and easy husbandry, rapid life cycle, and many available genetic tools [5, 10–16]. These advantages contribute to the status of *Drosophila* as a premier model for reverse-engineering multicellular systems. Of note, several fundamental signaling pathways were first discovered in *Drosophila*, including Hedgehog [17], Notch [18] and Wingless pathways [19]. Therefore, *Drosophila* has been extremely crucial in biology and bioengineering researches in many areas and will surely continue to play a critical role in years to come [20].

Beyond fundamental research, *Drosophila* has been used to study many health challenges, including cancer [21–28], neurodegenerative disorders [29–31], infectious diseases [32], cardiac disease [33], aging and metabolic diseases [34], wound healing and organ regeneration [20, 35–38] (Fig. 1b). *Drosophila* disease models can accelerate the rate of therapeutic drug testing and discovery due to the availability of genetic tools and a genome that lacks redundancy [11, 39–41]. Thus, *Drosophila* has a proven track record for understanding the biocomplexity of multicellular systems.

Here, we review a selected set of engineering tools and methodologies that are broadly applicable to reverse-engineer organ development. As a case in point, we focus on selected examples centered on the quantitative analysis of *Drosophila* (Fig. 1). This review highlights selected engineering advances that have led to the development of tools in the field of high-throughput and high-content screening: microfluidic devices, imaging technologies, and imaging analysis algorithms. Many novel and elegant engineering designs, such as various microfluidic devices and imaging modalities, have more precise manipulations and extract deeper insights from genetic systems, with a large breadth applied to the zebrafish, the fruit fly and the worm [42–45]. Rapid advances in machine learning and deep learning have greatly increased researchers’ ability to extract and analyze biological data. These tools are enabling increasingly quantitative characterization of fruit flies and other multicellular systems. Finally, the availability of many computational modeling tools (see, for example, reviews such as [46, 47]) has facilitated and accelerated the iterative cycle of hypothesis testing and revision (Fig. 1a). The review concludes with a perspective on current trends and future potential directions for reverse-engineering of multicellular systems.

## Microfluidic devices enable controlled imaging and perturbations of fruit fly development

Microfluidic devices refer to systems that use channels with dimensions of tens to hundreds of micrometers to manipulate a small amount of fluids [48]. A big challenge in studying the fruit fly is how to accurately apply perturbations and manipulate its organs due to their small size. Microfluidic devices are an increasingly important technique for addressing this challenge. In the following section, we discuss how microfluidic devices were applied in representative individual studies and how they have contributed to the improvement of current experimental approaches.

### Sample preparation and immobilization

Immobilization is a critical step to achieve high resolution imaging and precise manipulation for moving samples, such as *Drosophila* larvae. For example, to study the larval nervous system, researchers require the larva to be immobilized to image neuronal physiological activities. However, immobilization of larvae is difficult because of its digging and burrowing motion. Traditional immobilization techniques, such as tape or glue, still allow minor larval movement and reduce larval viability [49, 50]. Therefore, several strategies have been developed to immobilize samples. For example, Mondal et al. used a deformable membrane controlled by a water column to mechanically restrain larvae. The device allows them to image vesicle trafficking in the neurons of *Drosophila*, *C. elegans*, and zebrafish at high resolution [51, 52]. Another chip designed by the same group immobilizes larvae by clamping the mouth region to reduce digging movement. There is an additional design that pneumatically immobilizes larvae and allows for automated larva loading, immobilization and unloading. Both methods achieved significant immobilization and resulted in high-resolution imaging of neural responses [53, 54]. Mechanical restraint achieves easy immobilization but leads to reduced viability and innate response to mechanical perturbation [53, 54].

**Fig. 2:**
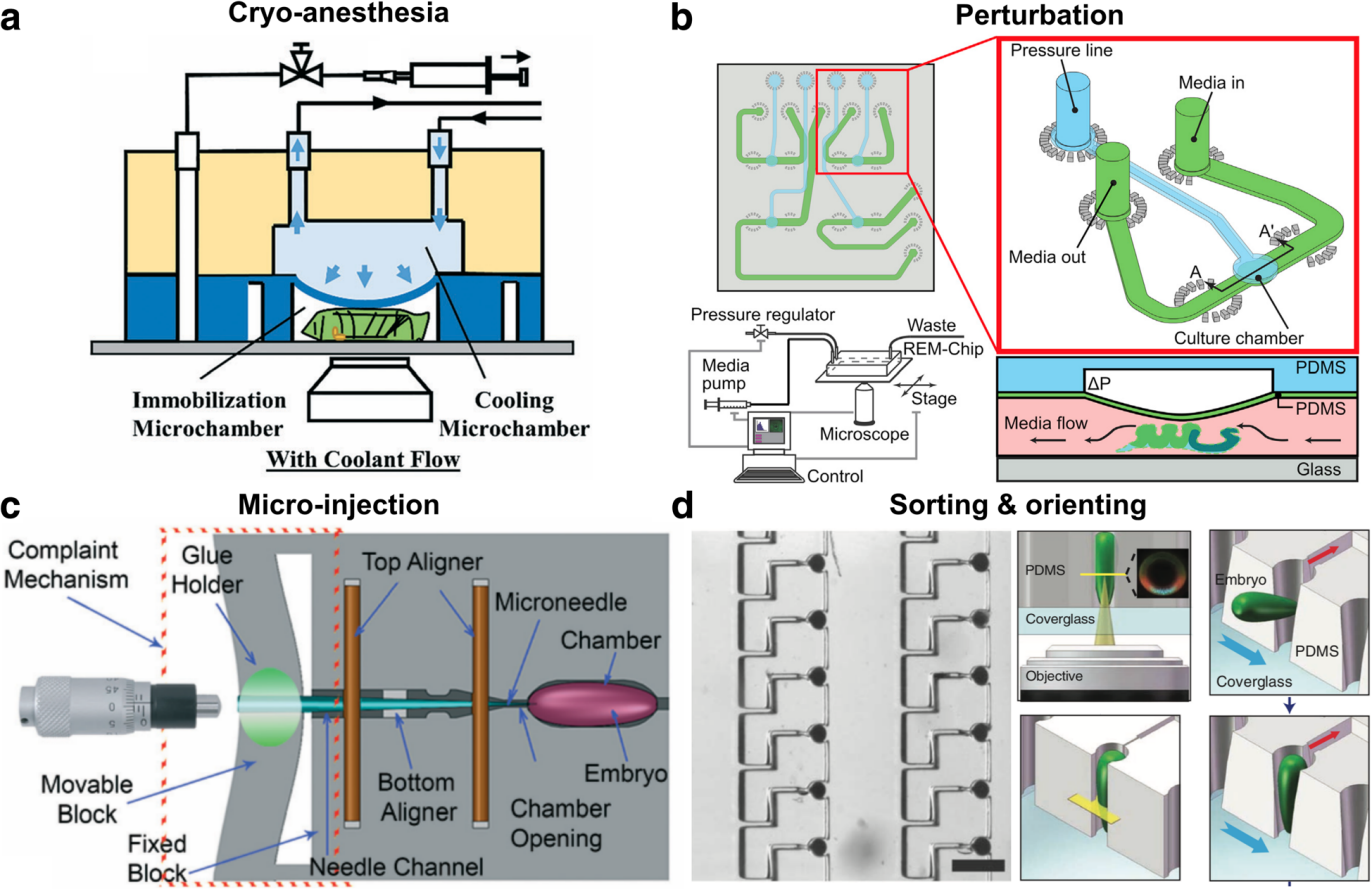
Microfluidic devices for handling, imaging and perturbing *Drosophila.* **a** Cryo-anesthesia presents an alternative to immobilization of larvae by physical restraint. The cryo-anesthesia device can support long-term observation while not affecting normal larval physiology. Figure modified with permission from [57]. **b** The REM-Chip is a device that precisely controls mechanical perturbation on *Drosophila* wing discs and couples chemical with mechanical perturbations. The device can be extended to integrate additional modalities, such as the application of electric fields. Figure modified with permission from [77]. **c** The automated microinjector allows more precise injection of genetic construct or drugs into the embryo in terms of location (5 μm resolution) and volume (as small as 30 pL) than existing microinjectors. Figure modified with permission from [61]. **d** The embryo-trap array rapidly orders and orients hundreds of *Drosophila* embryos in a high-throughput manner, permitting systematic study of dorsoventral development of the embryo. It enables parallel imaging of dorsoventral plane in hundreds of embryos. Figure modified with permission from [67]

Anesthesia is an alternative to mechanical immobilization. Heemskerk et al. developed an immobilization chamber that uses desflurane for anesthesia [55]. A newer design uses both CO_2_ and compression to immobilize larvae [56]. The chip also incorporates inputs for food feeding that allow for long-term (> 10 h) immobilization and imaging. Researchers were able to observe regenerative axonal growth up to 11 h of injury of the larva, demonstrating that CO_2_ did not affect the physiology of the larva in this study. An improved design uses coolant, instead of CO_2_, for anesthesia and immobilization (Fig. 2a). This technique enabled the imaging of in vivo mitochondria movement in axons with high resolution without affecting the larva physiology [57].

Orienting a multicellular sample during loading is a frequently encountered problem. To overcome this, Ardeshiri et al. employed a rotatable glass that can suck onto the head of the larva to rotate the larva [49, 58]. Another creative solution allows samples to be prepared on the cover glass first before the silicone slab is placed on top to form the channels of the device [59]. This design allows more flexible preparations, better orientations and wider accommodation of a variety of samples.

### Microinjection

Delivery of genetic constructs into fly embryos requires precise microinjection. For perturbation studies, drugs/toxins must also be accurately introduced into fragile embryos. Due to the requirement of precise placement and the small volume of injection, microinjectors have become tools of choice. Several microfluidic devices have been created to miniaturize this technique and to surpass the reliability of manual injection. First, Delubac et al. designed a microfluidic system for automatic embryo loading, detection and injection [60]. The device retrieves and places the embryos in contact with the injector/needle. The injection begins when the system detects the embryo in front of the injector. This fully-automated process enables high-throughput screening of embryos and/or creation of transgenic *Drosophila* lines. However, there is no control as to how deep the injector can go. Later, Ghaemi et al. incorporated a long-taper needle and a micro-positioner to control the depth of injection (Fig. 2c) [61]. This system enables deep (up to 250 μm), highly-precise injections (a resolution of 5 μm) and low injection volumes (as low as 30 ± 10 pL) with minimum damage because of the tapered needle. The precise (position and volume) injection of toxins (NaN_3_) into specific locations of the *Drosophila* embryo enables a detailed spatiotemporal study of how toxins affect embryo development [61].

### Sorting, positioning and orienting of samples

One of the advantages of using *Drosophila* embryos is the high-throughput data collection enabled by the number of embryos that can be obtained at low cost. However, sorting, positioning and orienting of many embryos or other post-embryonic organs is a technical hurdle that needs to be addressed. Furlong et al. adopted the concept of fluorescence-activated cell sorting (FACS) and designed a device for sorting embryos expressing a fluorescent protein marker [62]. The device uses a robotic valve to separate the embryos into fluorescent and non-fluorescent samples. In 2004, Chen et al. presented a pressure-controlled microfluidic sorter for *Drosophila* embryos that directs the flow direction of embryos into different outlets [63]. The computer simulation and flow experiment with dye demonstrated the functionality of the device. Chen et al. improved the design to allow for high-speed sorting, enabled by a deflecting jet to change the movement of the object [64].

Bernstein et al. presented an early attempt to position and orient *Drosophila* embryos in batch for high-throughput microinjection. They designed a micro-assembly of protruded hydrophobic surfaces to achieve large-scale positioning and orienting of the embryos [65]. Embryos are flowed through the device and are immobilized when in contact with the hydrophobic surface. The designed achieved 95% immobilization rate and 40% alignment rate. They also presented a conceptual design of the high-throughput microinjection system that would work with the orientation array, still yet to be realized as a physical working model [66].

Lu and collaborators developed a series of array-based microfluidic devices for positioning and orienting *Drosophila* embryos. A first microfluidic array was designed to utilize passive hydrodynamics to trap, position and vertically orient *Drosophila* embryos (Fig. 2d) [67, 68]. The vertical orientation of the embryo allows the observation of dorsal-ventral patterning of proteins of interest. The device provided high-throughput dorsoventral patterning data. Subsequently, the researchers modified the device to horizontally orient the embryo [69]. The Lu lab further improved the design to increase the loading efficiency to > 90% [70]. The new iteration also allows for anoxia perturbation of the embryos and potentially other forms of perturbation.

### Multi-modal perturbations to organ systems

Spatiotemporal control over a range of perturbations (e.g. mechanical, chemical and electrical) on multicellular samples often requires multi-modal microfluidic device designs. Lucchetta et al. designed pioneering microfluidic devices to investigate how temperature regulates embryogenesis [71, 72]. The device generates a temperature step between the two compartments of a *Drosophila* embryo. This spatiotemporal perturbation of temperature created a way to understand the complex biochemical networks governing *Drosophila* embryogenesis [73]. Researchers have adopted this design and used it for other perturbations. For example, a similar design exerts spatiotemporal control of oxygen gradient on living embryos [74]. To accommodate various *Drosophila* samples and apply different kinds of chemical stimuli, Giesen et al. came up with a device that can immobilize a range of *Drosophila* organs and apply chemical stimulations [75]. The authors demonstrated the use of the device to perturb and image brain, leg and proboscis. They successfully measured calcium-based neuron responses to chemical stimuli at single-cell resolution using this device.

Zhang et al. devised a microfluidic system that applies millinewton-level mechanical stimuli to *Drosophila* larvae [76]. The system uses a pipette controlled by a robotic system to apply the mechanical stimulation. The robotic system significantly increases the accuracy and consistency of mechanical stimulation over manual operation. Another device that allows for precise mechanical perturbation of organs uses a diaphragm deflectable by pneumatic pressure to apply uniaxial compression on *Drosophila* wing disc (Fig. 2b) [77]. Using this device, Narciso et al. probed the genetic and mechanical mechanisms of Ca^2+^ signaling in wing discs, a model organ for investigating signal transduction during organ growth. The device allows accurate mechanical stimulation of the wing disc, and it can be modified to accommodate other organoid-size systems and/or adding additional perturbations, such as electric stimulation [78].

### Trends for microfluidic devices for multicellular systems

Microfluidic devices enable high-throughput analysis and perturbation with high spatiotemporal resolution. Recent efforts have combined functionalities that were traditionally achieved by multiple microfluidic devices into one design. For example, Shorr et al. invented a device that incorporates various automated operations of *Drosophila* embryo, including high-throughput automatic alignment, immobilization, compression, real-time imaging, and recovery of hundreds of live embryos [79]. These new devices have achieved multiplexing of various modalities, and allow for acceleration of research in developmental biology and multicellular systems [80].

The possibilities brought up by microfluidic devices are numerous and the development of new manufacturing technologies is helping the democratization of microfluidic devices as well. Computer-aided design (CAD) and simulation have greatly increased the accuracy and functionality of newly-designed devices [63, 64, 79]. 3D printing is enabling the customizable production of microfluidic chips [81, 82], as the resolution of those printers has improved significantly. 3D printers have brought down the cost of manufacturing and enabled the easy transfer of designs [80]. Other quick-fabrication techniques, such as hybrid-polyethylene-terephthalate laminate (PETL), are also lowering the barrier to entry for microfluidic devices [78, 83]. In addition, many universities are also providing training programs and have clean-room facilities that can support the adoption of microfluidic devices among new users [80]. Combined, these developments are encouraging the development of microfluidic devices with new applications in developmental biology and the synthetic biology of multicellular systems.

## Three-dimensional imaging modalities enable the analysis of thick multicellular systems

**Fig. 3:**
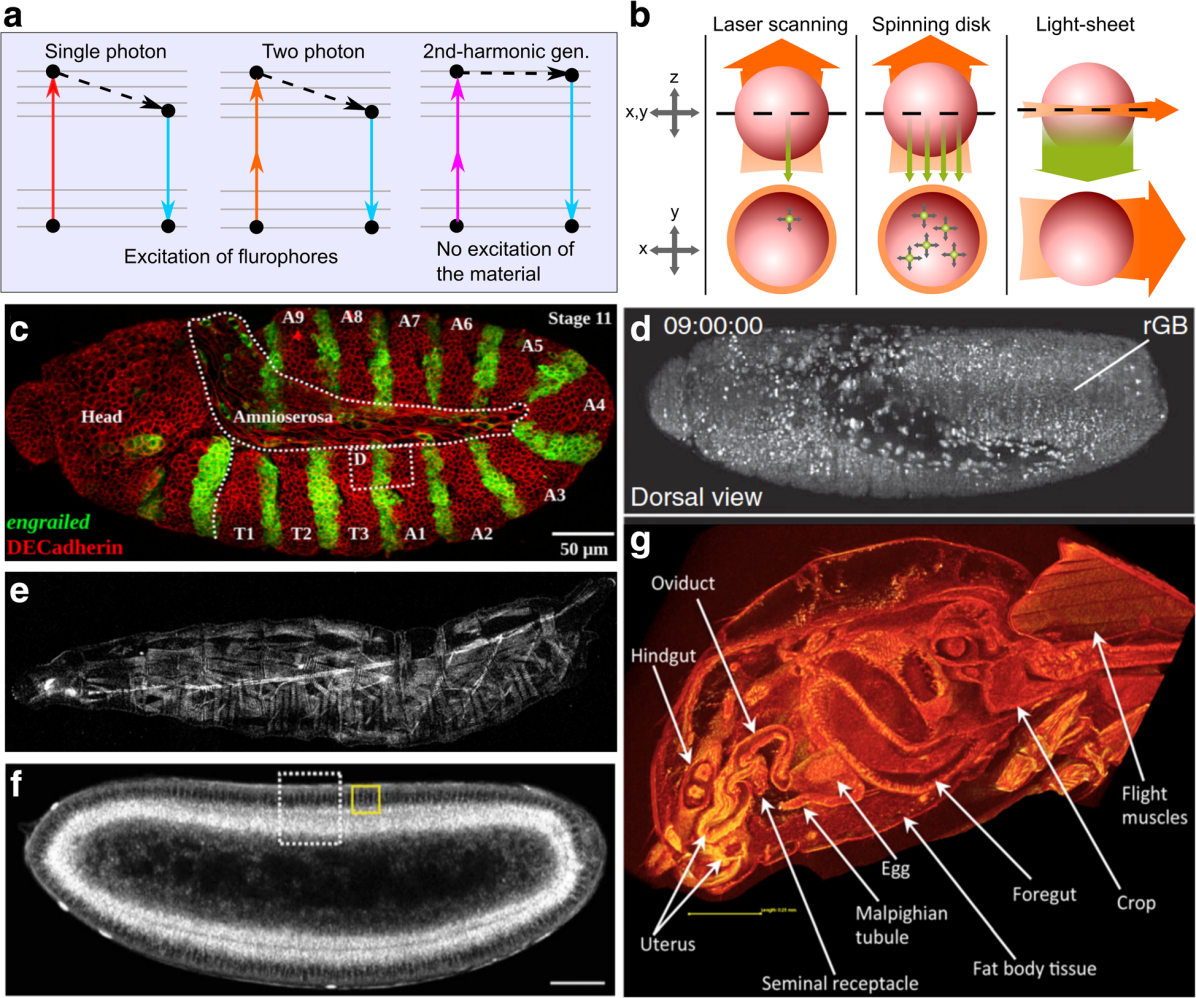
Imaging technologies open doors to deeper insights of *Drosophila.* **a** Single-photon (confocal) microscopy and multi-photon microscopy visualize samples by exciting the fluorophore and detect the emitted fluorescence. Harmonic generation microscopy, however, does not involve excitation of target molecules for visualization. Second-harmonic generation involves the combination of two photons into one photon without loss of energy. **b** Laser scanning confocal and spinning disk confocal microscopes illuminate the whole sample and detects epifluorescence, while light-sheet only illuminates the focal plane and detects fluorescence from the perpendicular direction. Adapted with permission from [196]. **c** Confocal microscopy can achieve excellent imaging quality for imaging tasks that do not require penetration deeper than 100 μm. Figure modified with permission from [197]. **d** SiMView combines two-photon microscopy with light-sheet microscopy that delivers high imaging speeds and near complete physical coverage of the embryo while reducing photobleaching and phototoxic effects. Scale bar: 50 μm. Figure modified with permission from [94]. **e** Second-harmonic generation microscopy visualizes muscular architecture and trachea system in detail without fluorophore labeling. Figure modified with permission from [112]. **f** Third-harmonic generation microscopy was used to visualize lipid trafficking. Scale bar: 50 μm. Figure modified with permission from [113]. **g** Micro-CT reveals the postmating responses by *Drosophila* female reproductive tract. Figure modified with permission from [125]

Due to the larger scales involved, multicellular systems, including *Drosophila* tissues, require three-dimensional imaging techniques. An increasingly diverse range of imaging modalities is enabling researchers to investigate deeper into tissues. Recent improvements of fluorescence-based imaging modalities have increased imaging resolution, sample penetration and acquisition rate while reducing phototoxicity and photobleaching [84, 85]. In the meantime, other new imaging modalities, such as harmonic generation microscopy and micro-computed tomography (micro-CT), enable label-free imaging [86, 87] (Fig. 3a, b). In this section, we discuss variations of fluorescent imaging techniques and label-free imaging. We also cover the advantages and limitations of each imaging modality.

### Confocal microscopy

Confocal microscopy uses a pinhole aperture to reject out-of-focus light to improve resolution and signal-to-noise ratio, compared to wide-field microscopy (Fig. 3c) [88]. Confocal microscopes can achieve a penetration depth of up to around 100 μm [89]. Confocal microscopy is divided into two main subcategories: laser scanning confocal microscopy and spinning disk confocal microscopy [89]. In laser scanning confocal microscopy, a single illumination spot is rastered across the field of view. The image acquisition rate is relatively low because of the point-by-point scanning system, especially when acquiring 3D stacks with multiple fluorescent channels from a sample. Because of the small focal point, laser scanning confocal microscopy can cause significant photobleaching and the specimen’s long-term viability is compromised due to phototoxicity [89]. Continuous efforts have resulted in significant increase of scanning speeds to lessen this limitation [90]. Alternatively, a spinning disk that contains many focus pinholes provides a multipoint scanning strategy that significantly increases the collection rate. This reduces photobleaching and improves specimen viability. However, this comes at a cost of reduced 3D-sectioning capability and resolution.

### Light-sheet fluorescent microscopy

In light-sheet microscopy, only a single plane of focus is illuminated (Fig. 3b). The camera detects fluorescence from a direction perpendicular to the light-sheet. The scanning speed of a light-sheet fluorescent microscopy is 100–1000 times faster than that of laser scanning confocal microscope. These characteristics minimize both phototoxicity and photobleaching and enable long-term imaging experiments of 3D multicellular systems [84]. This advantage allows imaging of a beating heart of a zebrafish or imaging of whole *Drosophila* embryos with fast rates of acquisition [91]. For example, *Drosophila* embryos can complete normal development even after being irradiated for 11,480 images by a light-sheet microscope [92]. The limited illumination of the specimen also results in high signal-to-noise ratio.

Light-sheet microscopes are highly customizable and can be coupled with other imaging techniques and/or downstream computational processing. For example, Greiss et al. achieved single-molecule imaging in a living *Drosophila* embryo, which is highly opaque in later stages, with reflected light-sheet microscopy [93]. Tomer et al. built a simultaneous multiview light-sheet microscopy that can acquire 175 million voxels per second (Fig. 3d) [94, 95]. Chhetri et al. developed isotropic multiview light-sheet microscopy for long-term imaging with double the penetration depth and 500-fold larger temporal resolution than previous design of light-sheet microscopes [96]. Aided by image segmentation and computational tracking, researchers reconstructed the geometry of the entire tissue and measured morphogenic dynamics during embryo development [97]. Lattice light-sheet microscopy, which results in an ultrathin light sheet, further increases the speed of image acquisition (scanning 200 to 1000 planes per second) with reduced phototoxicity [98].

Light-sheet microscopes can be constructed at relatively low cost, compared with other imaging technology setups. A great resource for building a customizable light-sheet microscope is an open hardware and software platform called OpenSPIM [99]. However, a significant challenge for light-sheet microscopes is how to process, store and move the very large datasets generated in single experiments.

### Multi-photon fluorescence microscopy

Multi-photon fluorescence microscopy relies on the simultaneous absorption of multiple photons to excite fluorophores (Fig. 3a). This process requires a high-energy laser concentrated at the laser focal point. Outside the focal point, the laser power is below the threshold required for two-photon excitation. This allows multi-photon microscopes to excite samples at a tiny volume around the focus point, thus reducing phototoxicity and extending the duration of in vivo imaging. The precise excitation at the focal point also improves the signal-to-noise ratio.

Multi-photon microscopes use near-infrared lasers with longer wavelengths (lower energy per photon) than lasers used in one-photon confocal microscopy. The near-infrared laser allows deeper penetration (2–3 times deeper for two-photon) into the sample, compared to confocal microscopy (Fig. 3d) [85]. The laser, because of the longer wavelength, also scatters less. Therefore, multi-photon microscopy provides good 3D sectioning capability for thick specimens. Researchers were able to image calcium dynamics in *Drosophila* adult brain in vivo in behavioral studies and odor-activated neuron response due to the deep penetration capability of two-photon microscopy, which is the most commonly used multi-photon microscopy [100–102]. Besides two-photon, three-photon microscopy has received increasing popularity because of its increased penetration and signal-to-noise ratio. For example, scientists have successfully imaged through adult mouse skulls at > 500 μm depth using three-photon microscopy [103].

However, multi-photon microscopy has low acquisition rates due to the point scanning system and leads to accelerated photobleaching [104, 105]. Two-photon microscopy also causes autofluorescence of some chromophores, such as NAD(P)H, which can cause significant noise for image acquisition [106]. The cost is also significantly higher because of the more sophisticated laser, optics, mechanics, and maintenance required. Nevertheless, the improvement of functionality and the continuous reduction of costs will enable multi-photon laser scanning microscopy to be adopted by the wider research community. Multi-photon microscopy currently defines the upper limit of penetration depth in diffraction-limited microscopy [85].

### Harmonic generation microscopy

The fluorescence microscopies discussed above have several innate shortcomings, such as photobleaching, phototoxicity, and the need to label the molecules [107]. Harmonic generation microscopy, on the other hand, achieves label-free imaging. Harmonic generation refers to the nonlinear optics phenomenon where multiple photons reach a molecule and generate a new photon without the presence of a fluorophore. For example, during second-harmonic generation, two identical incoming photons are combined to generate one outgoing photon with a wavelength of exactly half of the excitation beam (Fig. 3a).

The biggest advantage of harmonic generation microscopy is that it does not require labeling of the molecules of interest. Harmonic generation microscopy also substantially reduces photobleaching and phototoxicity because it does not rely on the excitation of fluorophores [108]. In addition, harmonic generation microscopy achieves deep penetration by using near-infrared wavelengths for the incident light. Harmonic generation microscopy has the ability to construct high-resolution three-dimensional images of several hundred microns of depth.

Harmonic generation provides additional structural information on molecular or supra-molecular order not easily detectable with fluorescence strategies. Second-harmonic generation is caused by materials that are noncentrosymmetric [109]. These materials include collagen fibril/fiber structure (type I and II fibrillar collagen), myofilaments, fibers, polarized microtubule assemblies, and muscle myosin (Fig. 3e) [87, 110–112]. Second-harmonic generation microscopy has been used to image developing muscle structures and the trachea system in 2nd-instar larva, and the lipid bodies in *Drosophila* cells [112, 113]. Researchers used second-harmonic generation microscopy to investigate the structure of *Drosophila* sarcomeres and visualize myocyte activity to study rhythmic muscle contraction [114, 115].

Third-harmonic generation occurs at structural interfaces with local transitions of the refractive index [116]. Third-harmonic generation was used to image lipid in *Drosophila* and mouse embryos. When coupled with second harmonic generation microscopy and two-photon imaging, one can explore the interactions between lipid, extracellular matrix and fluorescence-marked proteins (Fig. 3f) [113, 117–119]. Researchers used third-harmonic generation to visualize rhodopsin in the eye [120], and to measure the morphogenetic movement in *Drosophila* embryos by visualizing lipid droplets around cell nuclei and the interfaces of yolk structures [121]. Together, second- and third-harmonic generation microscopy modalities serve as powerful label-free imaging techniques.

### Micro-CT

Micro-computed tomography (micro-CT), like traditional CT, uses X-rays to produce sectioning of a sample and uses computers to reconstruct the 3D morphology of the specimen [122]. Micro-CT produces images with microscopic resolution and avoids artifacts due to processing of samples used for fluorescence imaging [123]. Because insects are made of only soft tissues, they are ideal for micro-CT. With very simple contrast staining, micro-CT can produce quantitative, high-resolution, high-contrast volume images of *Drosophila,* bumblebee, etc. [86, 124]. Micro-CT has become increasingly popular and is used to study morphological changes in a broad range of *Drosophila* tissues (Fig. 3g), including the female reproductive tract [125], neuronal structures [126], urolithiasis studies of calcium oxalate deposition [127], and wings for computational aerodynamic analysis [128].

The combination of multiple imaging modalities opens new possibilities to utilize the strengths while avoiding the limitations of individual techniques. For example, Truong et al. combined two-photon microscopy with light-sheet microscopy to implement two-photon-scanned light-sheet microscopy for *Drosophila* embryos [129]. This combination achieved twice the penetration of one-photon light-sheet microscopy and is more than ten times faster than two-photon laser scanning microscopy. Researchers also combined multi-photon microscopy with harmonic generation microscopy to construct a comprehensive picture of samples including both the fluorophore-labeled molecules and non-labeled structural molecules [130]. However, a major challenge for systems bioengineers is to process large datasets generated by these advanced imaging techniques. There is a critical need to automate the analysis of large datasets and to reduce high-dimensional data that includes information of molecular species and biophysical properties of cells through both space and time [131].

### Trends of imaging technologies for multicellular systems

Besides the introduction of new imaging principles, existing imaging technologies are often combined for multiplexing of functionalities that further increases in performance [93–96, 98]. There is also a trend of democratization of imaging technologies, from the OpenSPIM project supporting the construction of customized light-sheet microscopes to mobile phone-based microscopy [99, 132–134]. The increase in acquisition speed and resolution encourages the advance of image analysis methods to handle the ever-increasing amount of data generated from analysis of multi-cellular systems with *Drosophila* providing a versatile system for proof-of-concept studies.

## Data-driven learning algorithms accelerate the quantitative analysis of multicellular systems

**Fig. 4:**
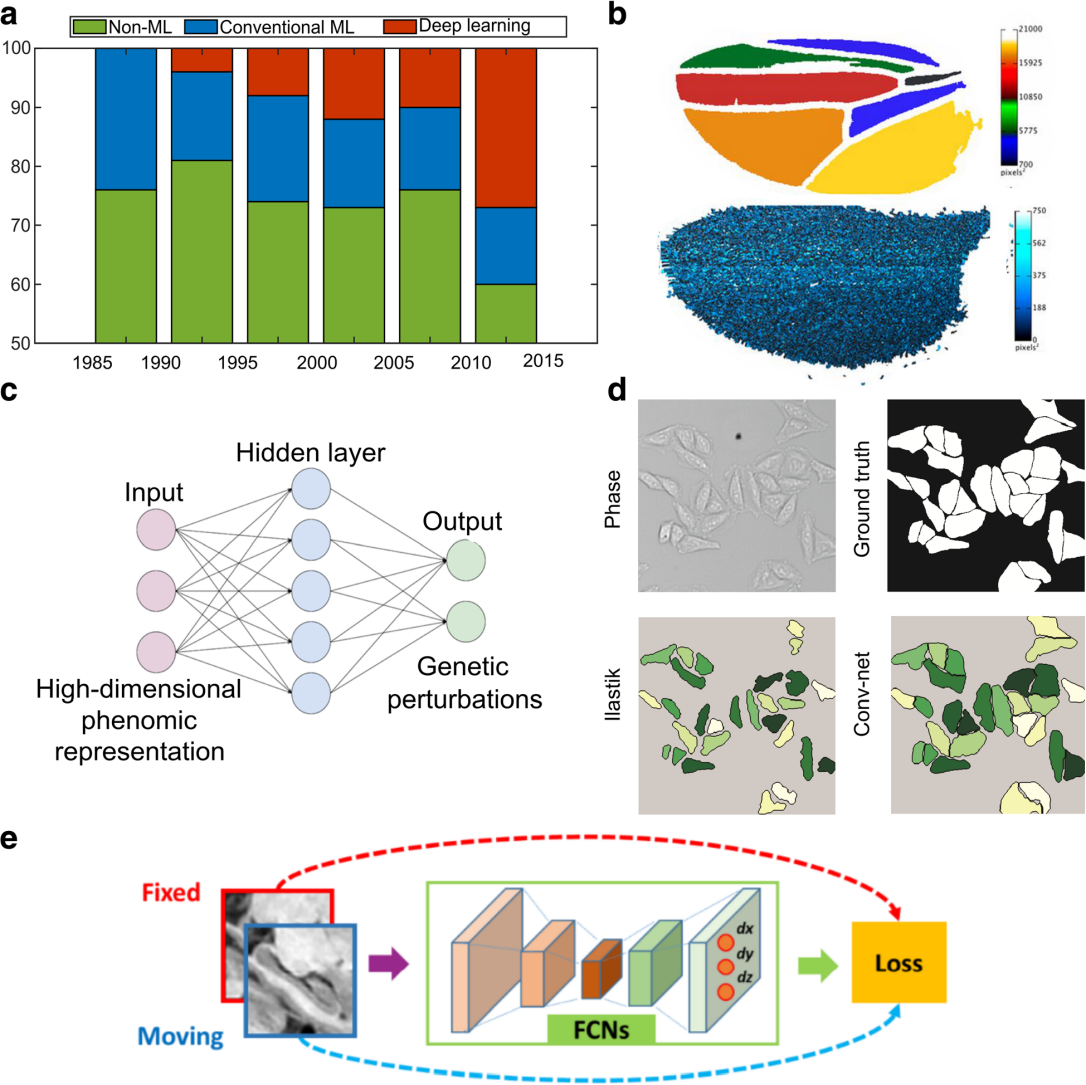
Data-driven learning accelerates the quantitative analysis in systems bioengineering. **a** The literature on cell image analysis shows an exponentially increasing interest in cell segmentation and the emergence of new approaches for this purpose. In total, 250 journal papers describing cell segmentation methods were analyzed in [198]. **b**) Upper panel shows automated extraction of trichrome densities for *Drosophila* wings using an open source package, FijiWings. Lower panel shows heat map of intervein area and trichrome densities for the whole wing blade using the same software. Figure modified with permission from [199]. **c** Schematic shows how the neural net architecture can be used for modelling many–one interactions between genetic perturbations and development. Figure modified with permission from [200]. **d** A comparison of segmentation methods demonstrates that convolutional neural network performs better than Ilastik (based on random forest) for segmentation of phase contrast images of HeLa cells. Figure modified with permission from [200]. **e** Schematic showing use of convolutional neural networks for the purpose of image registration. Figure modified with permission from [163]

The exponential increase in biological data acquisition rates challenges conventional analysis strategies [135]. Integration of advanced algorithms for bio-image analysis is thus highly desired. The result of a bio-image analysis pipeline can be as simple as quantification of fluctuations in cellular areas over time or as complex as a high-dimensional array of features of a *Drosophila* wing. In short, the goal of analysis is to convert images into arrays of numbers that are amenable to statistical evaluation. This helps create data-driven models or to validate predictions from phenomenological or mechanistic models. In this section, we discuss how both conventional machine-learning and deep-learning algorithms play critical roles in the analysis of multicellular systems, using selected examples focused on the fruit fly. In particular, we show how deep learning is rapidly emerging as a solution to accelerate the analysis of biological big data (Fig. 4a).

**Fig. 5:**
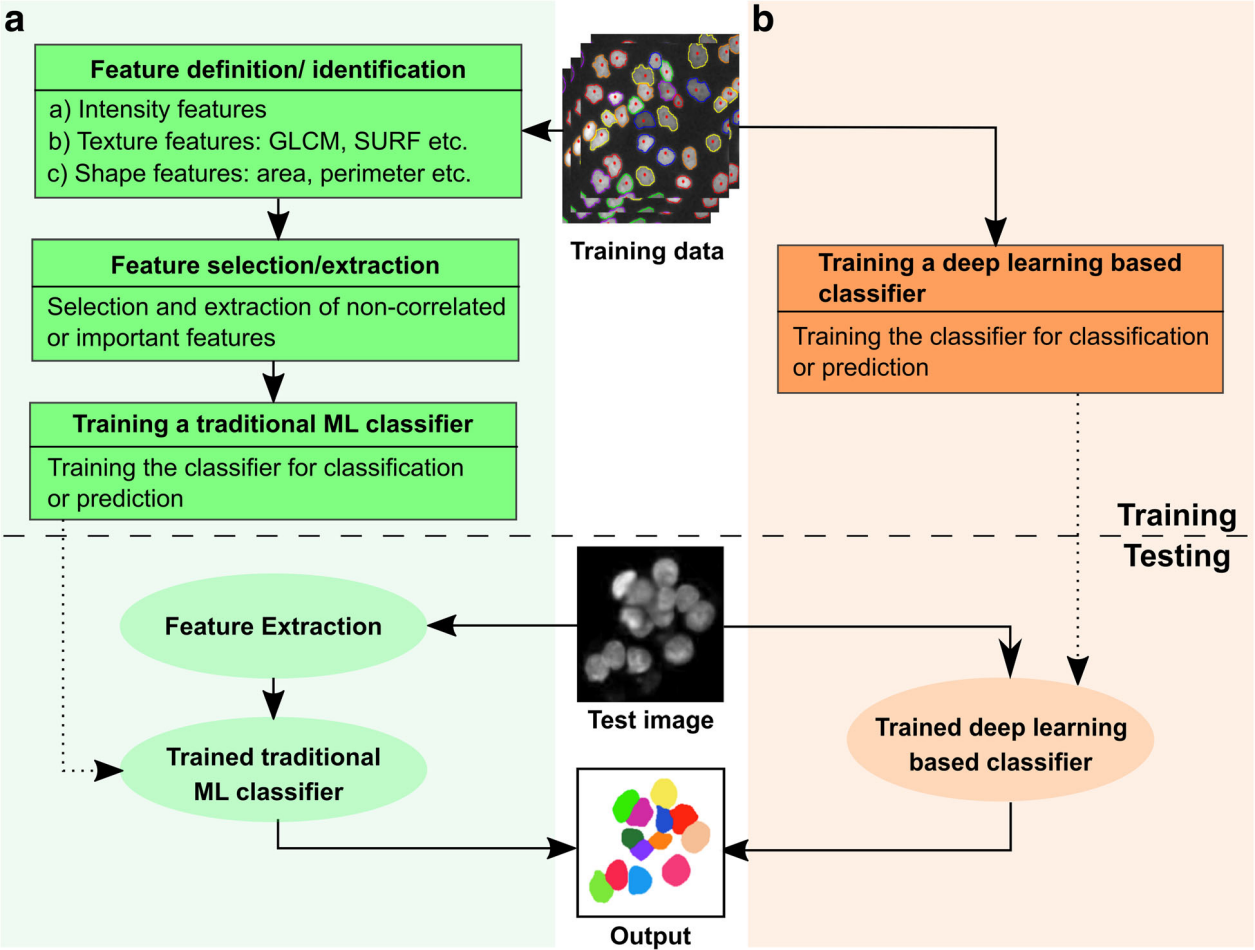
Workflow utilizing supervised machine learning for classification and prediction. **a** A supervised machine learning approach first requires the algorithm to learn the task of classification/prediction, based on the training data. Conventional machine learning approaches require another set of algorithms for identifying, selecting and extracting the features from the images. The extracted features are then used for projecting the image into a high-dimensional feature space. The task of classification/prediction is then done over this feature space. **b** In contrast, deep learning identifies these features through its complex neural architecture, trying to mimic the human brain, without requiring additional steps for it. Once trained, these models tend to perform much faster and are suitable for real-time quantification

Machine-learning algorithms leverage training datasets to find features within the data to fulfill the task of either classification or prediction [136]. A feature is a measurable property or characteristic of a phenomenon within the image. Feature extraction can either be manual or embedded within the algorithm’s architecture. Machine-learning algorithms are either supervised (requiring example input-output pairs to train the algorithm) or unsupervised (input data not annotated). Unsupervised learning algorithms, such as k-means clustering, perform poorly on noisy datasets and are frequently unsuited to bio-image analysis [137]. Therefore, supervised machine-learning algorithms are more commonly adopted for bio-image analysis (Fig. 5).

One of the major challenges in cellular tracking is obtaining high-quality segmentation masks of cells and separating regions of interest from noisy images at each time points. Non-machine-learning techniques, such as Otsu’s method [138] and P-tile method [139], are very sensitive to noise and do not produce good quality segmentation masks. An alternative approach is using region accumulation algorithms, such as watershed transformation [140]  as implemented in EpiTools [141], where seed points are defined within the image and are iteratively grown to form the complete label [142]. However, these algorithms result in over-segmentation and require further manual processing.

In comparison, researchers have started using supervised machine learning based on pixel classifiers for image segmentation because of their versatility and robustness. Some of the most widely used algorithms in designing a pixel classifier are support vector machines [143], adaptive boosting (AdaBoost) [144] and random forest [145]. A number of open-source packages, such as CellProfiler [146], Ilastik [147], CellCognition [148], PhenoRipper [149], Wndchrm [150], Fiji [151] and EBImage [152], implement the above algorithms. However, the algorithms used in most of the existing packages require selection of features by a user (Fig. 4b). Incorporating too many features slows down the implementation of the algorithm and makes them unsuitable for real-time quantification. Manual feature selection and extraction also increase the processing time for each image and hence make these algorithms unsuitable for big data processing.

To resolve these issues, researchers have started to use a class of machine learning algorithms called deep learning, which completely bypasses manual feature extraction. Deep-learning techniques achieve higher accuracies than classical machine-learning methods. These algorithms rely on neural networks, where layers of neuron-like nodes mimic how human brains analyze information (Fig. 4c) [153]. Since deep learning is a relatively new concept in computer vision, its impact in the field of bio-image informatics is yet to be fully realized [154]. The architecture of neural networks automates the extraction of features, thus eliminating the need for feature selection (Fig. 5). Thus, deep-learning algorithms are suitable for processing large datasets as there is a significant reduction in computational time achieved by avoiding a separate task of feature extraction. Once trained, deep-learning algorithms can analyze data from new sources of bio-images.

Rapid development in processing capabilities and availability of packages, such as TensorFlow [155], Blocks and Fuel [156], Torch [157], Caffe [158] and MATLAB, are making deep-learning techniques widely accessible to the systems biology and bioengineering communities. Deep-learning algorithms generate more accurate segmentation masks in less time, compared to conventional supervised learning algorithms.

One of the most common deep-learning algorithms is convolutional neural network (CNN) [159]. In a CNN, every network layer acts as a detection filter for the presence of specific patterns in the data. The first layers in a CNN detect large patterns that can be recognized and interpreted relatively easily. Later layers detect increasingly smaller patterns that are more abstract. The last layer makes an ultra-specific classification by combining all the specific patterns detected by the previous layers. However, the usage of this class of algorithms is heavily restricted by the amount of training data available in biology. To overcome this problem, a modified full CNN called U-Net was created [160]. U-Net was used to segment cells in *Drosophila* first instar larva ventral nerve cord using only 30 training images, thus significantly reducing the size of training data required for conventional CNN. Duan et al. used CNN to identify and mark the heart region of *Drosophila* at different developmental stages [161]. The algorithm performs better than the conventional machine-learning algorithms (Fig. 4d).

Additional applications of deep learning for analyzing multicellular systems in *Drosophila* include image registration. For example, cultured samples often move during image acquisition. The movement, along with deformations within the tissue, makes spatial quantification of features a difficult task. Image registration for biological samples is a two-step process: a) segmentation to identify regions to be registered, and (b) registration of the region of interest. Conventional machine-learning algorithms are not well-suited for this task as they often rely on manual identification of intensity-based features that vary over time. Liang et al. used deep learning to segment out the pouch from time-lapse movies of *Drosophila* wing discs that expresses GCaMP6, a genetically-encoded fluorescent sensor [162]. Segmenting and registering the wing disc is challenging due to the highly dynamic and stochastic Ca^2+^ dynamics [162]. The full CNN architecture identifies high-level embedded patterns, which are sometimes impossible to identify and extract manually. Segmentation was followed by a modified traditional image registration approach for tracking the moving wing disc pouch. Similarly, a full CNN was also used with a novel non-rigid image registration algorithm to optimize and learn spatial transformations between pair of images to be registered (Fig. 4e) [163].

### Trends of data analysis techniques for multicellular systems

In summary, data-driven learning algorithms, such as machine learning and deep learning, serve as powerful new techniques for image processing of multicellular systems such as *Drosophila*. These algorithms can be used to tackle complicated problems and reveal structure in data that is too big or too complex for the human brain to comprehend. One of the biggest challenges in using these algorithms is that they require extremely large datasets that are well-annotated to train the algorithm. To circumvent this challenge, researchers have been working on ways to train models more efficiently with less data. Advancements in transfer learning enable the deep learning to apply classification capabilities acquired from one data type to another data type, thus increasing its robustness [164]. However, there are several challenges that need to be overcome to fully unleash the power of deep learning in biological research. A significant challenge is to make these techniques accessible. Collaborations are required between computer vision researchers and biologists for developing general-use packages. Support and proper documentation standards are needed for maintaining new computational packages to enable researchers to benefit and more quickly adopt new algorithm methodologies.

## Concluding perspectives

Systematic approaches that integrate advanced microfluidic devices, imaging acquisition, and machine learning are essential techniques for analyzing the development of multicellular systems. There is an emerging need and intensive focus toward accelerating the cycle of hypothesis generation and testing and interdisciplinary collaboration through the engineering of integrative experimental and computational pipelines (Fig. 1b). Significant progress is being made that combines device manufacturing, computer vision, statistical analysis with mechanical automation of time-consuming biological experiments by multidisciplinary teams [165, 166].

From the traditional fluorescence-based imaging to X-ray-based micro-CT, we are seeing a range of new imaging technologies being applied to multicellular systems, including genetic model systems such as *Drosophila*. Advances in traditional fluorescence-based imaging is also significantly increasing image-acquisition speed, penetration and signal-to-noise ratio [93, 95, 96, 102]. In the meantime, label-free imaging of structure and/or measurements of tissue mechanics is leading to broader applications [111, 167]. These imaging modalities further combine with other technologies to provide increasing imaging capabilities. An emerging bottleneck for automating multimodal imaging experiments is the need to develop capabilities for parallel imaging modules integrated with customizable multichannel microfluidic devices to image many biological samples at a time. This, in turn, will increase the need for data storage and management solutions for labs. The significant advances being made in acquisition speed and resolution also demands a paradigm shift of analysis methods to handle the gigabytes and terabytes of data that are generated per imaging session [94, 96]. These new trends are blurring the knowledge boundaries of different research disciplines and encouraging the collaboration of microfluidic device designers, imaging technicians and computer vision scientists.

With the large amount of image data generated from experiments, machine learning is becoming an integral part of bio-image analysis. Significant progress in terms of computational power and availability of open-source modeling languages like TensorFlow has made machine learning accessible to cell and developmental biologists. Recently developed algorithms, based on the concept of transfer learning, has decreased the required sample sizes needed for training learning algorithms. For instance, U-Net required only 30 training images to analyze *Drosophila* larval neural cord, compared with hundreds of images needed for traditional CNN [160]. Algorithms that perform even faster than U-Net, such as context encoding networks, Mask R-CNN and Deeplabv3+, have also been proposed recently [168–170]. However, a domain expert is required to implement these techniques, because they require fine-tuning of parameters and hyperparameters within the network [171]. Currently, computer vision algorithms can handle a variety of tasks, including registration of dynamic imaging data, removal of obstructing elements in images, normalization of images, improvement of image quality, repair of data, and pattern discovery [172–174]. These algorithms will enable more robust and accurate quantification of images of multicellular systems.

Finally, computational models are an additional tool for reverse-engineering multicellular systems. They are often required to generate new insights for explaining emergent phenomena. They also systematize the process of hypothesis generation to close the iterative loop in reverse-engineering multicellular systems (Fig. 1a). For example, the interplay between mechanical forces, biochemistry and genetics governs how cells organize themselves into organs (as reviewed in [6]). These processes require computational models to integrate experimental data and reduce the complexity to identify underlying principles governing system behavior [175]. Historically, *Drosophila* provides an ideal playground for developing and testing computational models of many aspects of development including pattern formation [176–180], organ growth control [181] and morphogenesis [182].

Various methods have been used to model cell-based processes in *Drosophila*, with a significant focus on modelling cell mechanics during morphogenesis. These methods include cellular Potts models, vertex models, continuum models, viscoelastic models, subcellular element models and immersed boudary methods, to name a few. Interested readers are referred to several reviews that focus on computational model development and validation [46, 47, 183]. A key consideration in analyzing multicellular systems is the need to account for heterogeneity (reviewed in [184]) and multiple length-scales (reviewed in [185, 186]). Another challenge is to develop multiscale models of physiological activities under different timescales, from milisecond to hours ([187], reviewed in [185, 188–190]). Finally, the integration of inference tools that estimate the subcellular distribution of forces is enabling more direct comparisons between model predictions and quantified experimental image-based data (one such example includes [191]). A couple of recent reviews on inference tools include [192–194].

A future goal for the reverse engineering of multicellular system should be the integration of data acquisition and analysis as highlighted in this review with the development and validation of computational models to guide the analysis of multicellular systems into generalizable pipelines [46]. Because of the variability of the experimental data in biology, there is a need to integrate uncertainty into model development. A Bayesian probabilistic framework is one mathematical strategy that incorporates uncertainty quantification into the optimization processes [195]. A Bayesian probabilistic framework can be used as a tool for estimating the parameters required to run bioprocess simulations, using experimental data extracted from bio-image analysis. Using such frameworks for biological systems will help in the robust and accurate quantification of parameters involved in computational simulations. In conclusion, the integrative engineering analysis of multicellular systems, often with *Drosophila* and other genetic model systems paving the way, is now reaching an exponential phase of synergistic growth.

## Abbreviations

AdaBoost: Adaptive boosting
CNN: Convolutional neural network
FACS: Fluorescence-activated cell sorting
Micro-CT: Micro-computed tomography
SEM: Subcellular element model

## References

[1] Nicholson JK, Holmes E, Lindon JC, Wilson ID (2004). The challenges of modeling mammalian biocomplexity. Nat Biotechnol.

[2] Michener WK, Baerwald TJ, Firth P, Palmer MA, Rosenberger JL, Sandlin EA (2001). Defining and unraveling biocomplexity. BioScience..

[3] Kitano H (2002). Systems Biology: A Brief Overview Science.

[4] Janes KA, Chandran PL, Ford RM, Lazzara MJ, Papin JA, Peirce SM (2017). An engineering design approach to systems biology. Integr Biol.

[5] Narciso C, Zartman J (2018). Reverse-engineering organogenesis through feedback loops between model systems. Curr Opin Biotechnol.

[6] Heisenberg C-P, Bellaïche Y (2013). Forces in tissue morphogenesis and patterning. Cell..

[7] Oates AC, Gorfinkiel N, González-Gaitán M, Heisenberg C-P (2009). Quantitative approaches in developmental biology. Nat Rev Genet.

[8] Hariharan IK (2015). Organ size control: lessons from Drosophila. Dev Cell.

[9] Edgar BA (2006). How flies get their size: genetics meets physiology. Nat Rev Genet.

[10] Bilen J, Bonini NM (2005). Drosophila as a model for human neurodegenerative disease. Annu Rev Genet.

[11] Pandey UB, Nichols CD (2011). Human disease models in Drosophila melanogaster and the role of the Fly in therapeutic drug discovery. Pharmacol Rev.

[12] Matthews KA, Kaufman TC, Gelbart WM (2005). Research resources for *Drosophila*: the expanding universe. Nat Rev Genet.

[13] St Johnston D (2002). The art and design of genetic screens: Drosophila melanogaster. Nat Rev Genet.

[14] Adams MD, Sekelsky JJ (2002). From sequence to phenotype: reverse genetics in *drosophila melanogaster*. Nat Rev Genet.

[15] Harding K, White K (2018). Drosophila as a model for developmental biology: stem cell-fate decisions in the developing nervous system. J Dev Biol.

[16] Hales KG, Korey CA, Larracuente AM, Roberts DM (2015). Genetics on the Fly: a primer on the Drosophila model system. Genetics..

[17] Nüsslein-Volhard C, Wieschaus E (1980). Mutations affecting segment number and polarity in Drosophila. Nature..

[18] Wharton KA, Johansen KM, Xu T, Artavanis-Tsakonas S (1985). Nucleotide sequence from the neurogenic locus notch implies a gene product that shares homology with proteins containing EGF-like repeats. Cell..

[19] Wodarz A, Nusse R (1998). Mechanisms of Wnt signaling in development. Annu Rev Cell Dev Biol.

[20] Jennings BH (2011). Drosophila – a versatile model in biology & medicine. Mater Today.

[21] Das T, Cagan R (2010). Drosophila as a novel therapeutic discovery tool for thyroid cancer. Thyroid..

[22] Fukushiro-Lopes DF, Hegel AD, Rao V, Wyatt D, Baker A, Breuer E-K (2017). Preclinical study of a Kv11.1 potassium channel activator as antineoplastic approach for breast cancer. Oncotarget..

[23] Breuer E-K, Fukushiro-Lopes D, Dalheim A, Burnette M, Zartman J, Kaja S (2019). Potassium channel activity controls breast cancer metastasis by affecting β-catenin signaling. Cell Death Dis.

[24] Vidal M, Cagan RL (2006). Drosophila models for cancer research. Curr Opin Genet Dev.

[25] Gladstone M, Su TT (2011). Chemical genetics and drug screening in Drosophila cancer models. J Genet Genomics.

[26] Brumby AM, Richardson HE (2005). Using *Drosophila melanogaster* to map human cancer pathways. Nat Rev Cancer.

[27] Pagliarini RA, Xu T (2003). A genetic screen in Drosophila for metastatic behavior. Science..

[28] Miles WO, Dyson NJ, Walker JA (2011). Modeling tumor invasion and metastasis in Drosophila. Dis Model Mech.

[29] Moloney A, Sattelle DB, Lomas DA, Crowther DC (2010). Alzheimer’s disease: insights from Drosophila melanogaster models. Trends Biochem Sci.

[30] Chan HYE, Bonini NM (2000). Drosophila models of human neurodegenerative disease. Cell Death Differ.

[31] Whitworth AJ. 1-Drosophila Models of Parkinson’s Disease. Adv Genet. 2011:1–50 Available from: http://www.sciencedirect.com/science/article/pii/B978012380860800001X. [cited 12 Oct 2018]. 10.1016/B978-0-12-380860-8.00001-X 21310293

[32] Dionne MS, Schneider DS (2008). Models of infectious diseases in the fruit fly Drosophila melanogaster. Dis Model Mech.

[33] Palandri A, Martin E, Russi M, Rera M, Tricoire H, Monnier V (2018). Identification of cardioprotective drugs by medium-scale in vivo pharmacological screening on a Drosophila cardiac model of Friedreich’s ataxia. Dis Model Mech.

[34] Owusu-Ansah E, Perrimon N (2014). Modeling metabolic homeostasis and nutrient sensing in Drosophila: implications for aging and metabolic diseases. Dis Model Mech.

[35] Bergantiños C, Vilana X, Corominas M, Serras F (2010). Imaginal discs: renaissance of a model for regenerative biology. BioEssays..

[36] Narciso C, Wu Q, Brodskiy P, Garston G, Baker R, Fletcher A (2015). Patterning of wound-induced intercellular ca 2+ flashes in a developing epithelium. Phys Biol.

[37] Matsubayashi Y, Millard TH (2014). Analysis of the molecular mechanisms of Reepithelialization in Drosophila embryos. Adv Wound Care.

[38] Brodskiy PA, Wu Q, Soundarrajan DK, Huizar FJ, Chen J, Liang P (2019). Decoding calcium signaling dynamics during Drosophila wing disc development. Biophys J.

[39] Chintapalli VR, Wang J, Dow JAT (2007). Using FlyAtlas to identify better *Drosophila melanogaster* models of human disease. Nat Genet.

[40] Fernández-Hernández I, Scheenaard E, Pollarolo G, Gonzalez C (2016). The translational relevance of Drosophila in drug discovery. EMBO Rep.

[41] Kasai Y, Cagan R (2010). Drosophila as a tool for personalized medicine: a primer. Personalized Med.

[42] Kamili F, Lu H (2018). Recent advances and trends in microfluidic platforms for C. elegans biological assays. Annual Rev Anal Chem.

[43] Hwang H, Lu H (2013). Microfluidic tools for developmental studies of small model organisms –nematodes, fruit flies, and zebrafish. Biotechnol J.

[44] Yang F, Gao C, Wang P, Zhang G-J, Chen Z (2016). Fish-on-a-chip: microfluidics for zebrafish research. Lab Chip.

[45] Kim AA, Nekimken AL, Fechner S, O’Brien LE, Pruitt BL. Chapter 12 - Microfluidics for mechanobiology of model organisms. In: Doh J, Fletcher D, Piel M, editors. Methods in Cell Biology: Academic Press; 2018. p. 217–59. Available from: http://www.sciencedirect.com/science/article/pii/S0091679X18300633. [cited 18 Nov 2018]. 10.1016/bs.mcb.2018.05.010 PMC6418080 30037463

[46] Fletcher AG, Cooper F, Baker RE. Mechanocellular models of epithelial morphogenesis. Philos Trans R Soc Lond B Biol Sci. 2017:372 Available from: https://www.ncbi.nlm.nih.gov/pmc/articles/PMC5379025/. [cited 14 Dec 2018]. 10.1098/rstb.2015.0519 PMC5379025 28348253

[47] Wyczalkowski MA, Chen Z, Filas BA, Varner VD, Taber LA (2012). Computational models for mechanics of morphogenesis. Birth Defects Res C.

[48] Whitesides GM. The origins and the future of microfluidics. Nature. 2006; Available from: http://www.nature.com/articles/nature05058. [cited 15 Oct 2018]. 10.1038/nature05058 16871203

[49] Ardeshiri R, Hosseini L, Amini N, Rezai P (2016). Cardiac screening of intact Drosophila melanogaster larvae under exposure to aqueous and gaseous toxins in a microfluidic device. RSC Adv.

[50] Restrepo S, Basler K (2016). Drosophila wing imaginal discs respond to mechanical injury via slow InsP3R-mediated intercellular calcium waves. Nat Commun.

[51] Mondal S, Ahlawat S, Rau K, Venkataraman V, Koushika SP (2011). Imaging in vivo neuronal transport in genetic model organisms using microfluidic devices. Traffic..

[52] Mondal S, Ahlawat S, Koushika SP. Simple Microfluidic Devices for in vivo Imaging of *C. elegans*, Drosophila and Zebrafish. J Vis Exp. 2012:3780 Available from: https://www.ncbi.nlm.nih.gov/pmc/articles/PMC3490237/. [cited 2 Sep 2018]. 10.3791/3780 PMC3490237 23051668

[53] Ghaemi R, Rezai P, Iyengar BG, Selvaganapathy PR (2015). Microfluidic devices for imaging neurological response of Drosophila melanogaster larva to auditory stimulus. Lab Chip.

[54] Ghaemi R, Rezai P, Nejad FR, Selvaganapathy PR (2017). Characterization of microfluidic clamps for immobilizing and imaging of Drosophila melanogaster larva’s central nervous system. Biomicrofluidics..

[55] Heemskerk I, Lecuit T, LeGoff L (2014). Dynamic clonal analysis based on chronic in vivo imaging allows multiscale quantification of growth in the Drosophila wing disc. Development..

[56] Ghannad-Rezaie M, Wang X, Mishra B, Collins C, Chronis N (2012). Microfluidic chips for in vivo imaging of cellular responses to neural injury in Drosophila larvae. PLoS One.

[57] Chaudhury AR, Insolera R, Hwang R-D, Fridell Y-W, Collins C, Chronis N (2017). On chip cryo-anesthesia of Drosophila larvae for high resolution in vivo imaging applications. Lab Chip.

[58] Ardeshiri R, Rezai P (2016). Lab-on-chips for manipulation of small-scale organisms to facilitate imaging of neurons and organs.

[59] Fan A, Tofangchi A, Venecia MD, Saif T (2018). A simple microfluidic platform for the partial treatment of insuspendable tissue samples with orientation control. Lab Chip.

[60] Delubac D, Highley CB, Witzberger-Krajcovic M, Ayoob JC, Furbee EC, Minden JS (2012). Microfluidic system with integrated microinjector for automated Drosophila embryo injection. Lab on a Chip.

[61] Ghaemi R, Arefi P, Stosic A, Acker M, Raza Q, Jacobs JR (2017). A microfluidic microinjector for toxicological and developmental studies in Drosophila embryos. Lab Chip.

[62] Furlong EEM, Profitt D, Scott MP (2001). Automated sorting of live transgenic embryos. Nat Biotechnol.

[63] Chen CC, Zappe S, Sahin O, Zhang XJ, Fish M, Scott M (2004). Design and operation of a microfluidic sorter for Drosophila embryos. Sensors Actuators B Chem.

[64] Chen CC, Wang JS, Solgaard O (2006). Micromachined bubble-jet cell sorter with multiple operation modes. Sensors Actuators B Chem.

[65] Bernstein RW, Zhang X, Zappe S, Fish M, Scott M, Solgaard O (2004). Characterization of fluidic microassembly for immobilization and positioning of Drosophila embryos in 2-D arrays. Sensors Actuators A Phys.

[66] Bernstein RW, Scott M, Solgaard O. In: Ma Z, Jin G, Chen X, editors. BioMEMS for high-throughput handling and microinjection of embryos. Beijing; 2004. p. 67. Available from: http://proceedings.spiedigitallibrary.org/proceeding.aspx?doi=10.1117/12.584626. [cited 23 Oct 2018].

[67] Chung K, Kim Y, Kanodia JS, Gong E, Shvartsman SY, Lu H (2011). A microfluidic array for large-scale ordering and orientation of embryos. Nat Methods.

[68] Levario TJ, Zhan M, Lim B, Shvartsman SY, Lu H (2013). Microfluidic trap array for massively parallel imaging of *Drosophila* embryos. Nat Protoc.

[69] Goyal Y, Levario TJ, Mattingly HH, Holmes S, Shvartsman SY, Lu H (2017). Parallel imaging of Drosophila embryos for quantitative analysis of genetic perturbations of the Ras pathway. Dis Model Mech.

[70] Levario TJ, Zhao C, Rouse T, Shvartsman SY, Lu H (2016). An integrated platform for large-scale data collection and precise perturbation of live *Drosophila* embryos. Sci Rep.

[71] Lucchetta EM, Lee JH, Fu LA, Patel NH, Ismagilov RF (2005). Dynamics of *Drosophila* embryonic patterning network perturbed in space and time using microfluidics. Nature..

[72] Lucchetta EM, Munson MS, Ismagilov RF (2006). Characterization of the local temperature in space and time around a developing Drosophila embryo in a microfluidic device. Lab on a Chip.

[73] Lucchetta EM, Carthew RW, Ismagilov RF (2009). The Endo-siRNA pathway is essential for robust development of the Drosophila embryo. PLoS One.

[74] Wang Z, Oppegard SC, Eddington DT, Cheng J (2017). Effect of localized hypoxia on Drosophila embryo development. PLoS One.

[75] van Giesen L, Neagu-Maier GL, Kwon JY, Sprecher SG (2016). A microfluidics-based method for measuring neuronal activity in *Drosophila* chemosensory neurons. Nat Protoc.

[76] Zhang W, Sobolevski A, Li B, Rao Y, Liu X (2016). An automated force-controlled robotic micromanipulation system for Mechanotransduction studies of Drosophila larvae. IEEE Trans Autom Sci Eng.

[77] Narciso CE, Contento NM, Storey TJ, Hoelzle DJ, Zartman JJ (2017). Release of applied mechanical loading stimulates intercellular calcium waves in Drosophila wing discs. Biophys J.

[78] Levis MK, Kumar N, Apakian E, Moreno C, Hernandez U, Olivares A, et al. Rapid fabrication of hybrid PETL-glass microfluidic devices for combined live imaging and multimodal perturbations of multicellular systems. Biomicrofluidics. 2019. In press. 10.1063/1.5086671 PMC6486393 31065310

[79] Shorr AZ, Mustafa Sonmez U, Minden JS, LeDuc P. High-throughput mechanotransduction in Drosophila embryos with mesofluidics. Lab on a Chip. 2019; Available from: https://pubs.rsc.org/en/content/articlelanding/2019/lc/c8lc01055b. [cited 18 Feb 2019]. 10.1039/c8lc01055b 30778467

[80] Sonnen KF, Merten CA (2019). Microfluidics as an emerging precision tool in developmental biology. Dev Cell.

[81] Beauchamp MJ, Nordin GP, Woolley AT (2017). Moving from millifluidic to truly microfluidic sub-100-μm cross-section 3D printed devices. Anal Bioanal Chem.

[82] Gong H, Woolley AT, Nordin GP (2016). High density 3D printed microfluidic valves, pumps, and multiplexers. Lab Chip.

[83] Cosson S, Aeberli LG, Brandenberg N, Lutolf MP (2015). Ultra-rapid prototyping of flexible, multi-layered microfluidic devices via razor writing. Lab on a Chip.

[84] Power RM, Huisken J (2017). A guide to light-sheet fluorescence microscopy for multiscale imaging. Nat Methods.

[85] Ntziachristos V (2010). Going deeper than microscopy: the optical imaging frontier in biology. Nat Methods.

[86] Smith DB, Bernhardt G, Raine NE, Abel RL, Sykes D, Ahmed F (2016). Exploring miniature insect brains using micro-CT scanning techniques. Sci Rep.

[87] Campagnola PJ, Millard AC, Terasaki M, Hoppe PE, Malone CJ, Mohler WA (2002). Three-dimensional high-resolution second-harmonic generation imaging of endogenous structural proteins in biological tissues. Biophys J.

[88] Thorn K, Kellogg D (2016). A quick guide to light microscopy in cell biology. MBoC.

[89] Jonkman J, Brown CM (2015). Any way you slice it—a comparison of confocal microscopy techniques. J Biomol Tech.

[90] Choi S, Kim P, Boutilier R, Kim MY, Lee YJ, Lee H (2013). Development of a high speed laser scanning confocal microscope with an acquisition rate up to 200 frames per second. Opt Express.

[91] Schmied C, Tomancak P (2016). Sample preparation and mounting of Drosophila embryos for Multiview light sheet microscopy. Drosophila: methods and protocols.

[92] Huisken J, Swoger J, Bene FD, Wittbrodt J, Stelzer EHK (2004). Optical sectioning deep inside live embryos by selective plane illumination microscopy. Science..

[93] Greiss F, Deligiannaki M, Jung C, Gaul U, Braun D (2016). Single-molecule imaging in living Drosophila embryos with reflected light-sheet microscopy. Biophys J.

[94] Tomer R, Khairy K, Amat F, Keller PJ (2012). Quantitative high-speed imaging of entire developing embryos with simultaneous multiview light-sheet microscopy. Nat Methods.

[95] Krzic U, Gunther S, Saunders TE, Streichan SJ, Hufnagel L (2012). Multiview light-sheet microscope for rapid *in toto* imaging. Nat Methods.

[96] Chhetri RK, Amat F, Wan Y, Höckendorf B, Lemon WC, Keller PJ (2015). Whole-animal functional and developmental imaging with isotropic spatial resolution. Nat Methods.

[97] Khairy K, Lemon WC, Amat F, Keller PJ (2015). Light sheet-based imaging and analysis of early embryogenesis in the fruit Fly. Tissue morphogenesis: methods and protocols.

[98] Chen B-C, Legant WR, Wang K, Shao L, Milkie DE, Davidson MW (2014). Lattice light-sheet microscopy: imaging molecules to embryos at high spatiotemporal resolution. Science..

[99] Pitrone PG, Schindelin J, Stuyvenberg L, Preibisch S, Weber M, Eliceiri KW (2013). OpenSPIM: an open-access light-sheet microscopy platform. Nat Methods.

[100] Seelig JD, Chiappe ME, Lott GK, Dutta A, Osborne JE, Reiser MB (2010). Two-photon calcium imaging from head-fixed *Drosophila* during optomotor walking behavior. Nat Methods.

[101] Wang JW, Wong AM, Flores J, Vosshall LB, Axel R (2003). Two-photon calcium imaging reveals an odor-evoked map of activity in the Fly brain. Cell..

[102] Paoli M, Haase A (2018). In vivo two-photon imaging of the olfactory system in insects. Olfactory receptors: methods and protocols.

[103] Wang T, Ouzounov DG, Wu C, Horton NG, Zhang B, Wu C-H (2018). Three-photon imaging of mouse brain structure and function through the intact skull. Nat Methods.

[104] Rebollo E, Karkali K, Mangione F, Martín-Blanco E (2014). Live imaging in Drosophila: the optical and genetic toolkits. Methods..

[105] Ustione A, Piston DW (2011). A simple introduction to multiphoton microscopy. J Microsc.

[106] Huang S, Heikal AA, Webb WW (2002). Two-photon fluorescence spectroscopy and microscopy of NAD (P) H and Flavoprotein. Biophys J.

[107] Friedl P, Wolf K, Harms G, von Andrian UH (2007). Biological second and third harmonic generation microscopy. Curr Protoc Cell Biol.

[108] Campagnola P (2011). Second harmonic generation imaging microscopy: applications to diseases diagnostics. Anal Chem.

[109] Chu S-W, Chen I-H, Liu T-M, Sun C-K, Lee S-P, Lin B-L (2002). Nonlinear bio-photonic crystal effects revealed with multimodal nonlinear microscopy. J Microsc.

[110] Williams RM, Zipfel WR, Webb WW (2001). Multiphoton microscopy in biological research. Curr Opin Chem Biol.

[111] Chen X, Nadiarynkh O, Plotnikov S, Campagnola PJ (2012). Second harmonic generation microscopy for quantitative analysis of collagen fibrillar structure. Nat Protoc.

[112] Lin C-Y, Hovhannisyan VA, Wu J-T, Lin C-W, Chen J-H, Lin S-J (2008). Label-free imaging of *Drosophila* larva by multiphoton autofluorescence and second harmonic generation microscopy. J Biomed Opt.

[113] Débarre D, Supatto W, Pena A-M, Fabre A, Tordjmann T, Combettes L (2006). Imaging lipid bodies in cells and tissues using third-harmonic generation microscopy. Nat Methods.

[114] Greenhalgh C, Stewart B, Cisek R, Prent N, Major A, Barzda V. Dynamic investigation of Drosophila myocytes with second harmonic generation microscopy. Quebec City, 2006; 634308. Available from: http://proceedings.spiedigitallibrary.org/proceeding.aspx?doi=10.1117/12.706559. [cited 7 Oct 2018]

[115] Greenhalgh C, Prent N, Green C, Cisek R, Major A, Stewart B (2007). Influence of semicrystalline order on the second-harmonic generation efficiency in the anisotropic bands of myocytes. Appl Opt.

[116] Weigelin B, Bakker G-J, Friedl P (2016). Third harmonic generation microscopy of cells and tissue organization. J Cell Sci.

[117] Débarre D, Supatto W, Farge E, Moulia B, Schanne-Klein M-C, Beaurepaire E (2004). Velocimetric third-harmonic generation microscopy: micrometer-scale quantification of morphogenetic movements in unstained embryos. Opt Lett.

[118] Supatto W, Débarre D, Farge E, Beaurepaire E (2005). Femtosecond pulse-induced microprocessing of live Drosophila embryos. Med Laser Appl.

[119] Watanabe T, Thayil A, Jesacher A, Grieve K, Debarre D, Wilson T (2010). Characterisation of the dynamic behaviour of lipid droplets in the early mouse embryo using adaptive harmonic generation microscopy. BMC Cell Biol.

[120] Karunendiran A, Cisek R, Tokarz D, Barzda V, Stewart BA (2017). Examination of Drosophila eye development with third harmonic generation microscopy. Biomed Opt Express.

[121] Supatto W, Débarre D, Moulia B, Brouzés E, Martin J-L, Farge E (2005). In vivo modulation of morphogenetic movements in Drosophila embryos with femtosecond laser pulses. PNAS..

[122] du Plessis A, Broeckhoven C, Guelpa A, le Roux SG (2017). Laboratory x-ray micro-computed tomography: a user guideline for biological samples. Gigascience.

[123] Sombke A, Lipke E, Michalik P, Uhl G, Harzsch S (2015). Potential and limitations of X-ray micro-computed tomography in arthropod neuroanatomy: a methodological and comparative survey. J Comp Neurol.

[124] Metscher BD (2009). MicroCT for comparative morphology: simple staining methods allow high-contrast 3D imaging of diverse non-mineralized animal tissues. BMC Physiol.

[125] Mattei AL, Riccio ML, Avila FW, Wolfner MF (2015). Integrated 3D view of postmating responses by the Drosophila melanogaster female reproductive tract, obtained by micro-computed tomography scanning. PNAS..

[126] Mizutani R, Takeuchi A, Hara T, Uesugi K, Suzuki Y (2007). Computed tomography imaging of the neuronal structure of *Drosophila* brain. J Synchrotron Radiat.

[127] Chen W-C, Chen H-Y, Liao P-C, Wang S-J, Tsai M-Y, Chen Y-H (2018). Toward a new insight of calcium oxalate stones in Drosophila by micro-computerized tomography. Urolithiasis..

[128] Brandt J, Doig G, Tsafnat N (2015). Computational aerodynamic analysis of a micro-CT based bio-realistic fruit Fly wing. PLoS One.

[129] Truong TV, Supatto W, Koos DS, Choi JM, Fraser SE (2011). Deep and fast live imaging with two-photon scanned light-sheet microscopy. Nat Methods.

[130] Zoumi A, Yeh A, Tromberg BJ (2002). Imaging cells and extracellular matrix in vivo by using second-harmonic generation and two-photon excited fluorescence. PNAS..

[131] Trisnadi N, Altinok A, Stathopoulos A, Reeves GT (2013). Image analysis and empirical modeling of gene and protein expression. Methods..

[132] Contreras-Naranjo JC, Wei Q, Ozcan A (2016). Mobile phone-based microscopy, sensing, and diagnostics. IEEE J Sel Topics Quantum Electron..

[133] McLeod E, Wei Q, Ozcan A (2015). Democratization of nanoscale imaging and sensing tools using photonics. Anal Chem.

[134] Ozcan A (2014). Mobile phones democratize and cultivate next-generation imaging, diagnostics and measurement tools. Lab Chip.

[135] Camacho DM, Collins KM, Powers RK, Costello JC, Collins JJ (2018). Next-generation machine learning for biological networks. Cell..

[136] Angermueller C, Pärnamaa T, Parts L, Stegle O (2016). Deep learning for computational biology. Mol Syst Biol.

[137] Raykov YP, Boukouvalas A, Baig F, Little MA (2016). What to Do When K-Means Clustering Fails: A Simple yet Principled Alternative Algorithm. PLoS One.

[138] Xiong G, Zhou X, Ji L (2006). Automated segmentation of Drosophila RNAi fluorescence cellular images using deformable models. IEEE Trans Circuits Sys I.

[139] Al-amri SS, Kalyankar NV (2010). Image Segmentation by Using Thershod Techniques.

[140] Ng HP, Ong SH, Foong KWC, Goh PS, Nowinski WL (2006). Medical Image Segmentation Using K-Means Clustering and Improved Watershed Algorithm. 2006 IEEE Southwest Symposium on Image Analysis and Interpretation.

[141] Heller D, Hoppe A, Restrepo S, Gatti L, Tournier AL, Tapon N (2016). EpiTools: an open-source image analysis toolkit for quantifying epithelial growth dynamics. Dev Cell.

[142] Mehnert A, Jackway P (1997). An improved seeded region growing algorithm. Pattern Recogn Lett.

[143] Burges CJC (1998). A tutorial on support vector machines for pattern recognition. Data Min Knowl Disc.

[144] Viola P, Jones M. Rapid object detection using a boosted cascade of simple features. Proceedings of the 2001 IEEE Computer Society Conference on Computer Vision and Pattern Recognition CVPR 2001. Kauai: IEEE Comput Soc; 2001; I-511-I–518. Available from: http://ieeexplore.ieee.org/document/990517/. [cited 14 Oct 2018]

[145] Liaw A (2002). Wiener M. Classification and Regression by randomForest.

[146] Carpenter AE, Jones TR, Lamprecht MR, Clarke C, Kang IH, Friman O (2006). CellProfiler: image analysis software for identifying and quantifying cell phenotypes. Genome Biol.

[147] Sommer C, Straehle C, Kothe U, Hamprecht FA (2011). Ilastik: Interactive learning and segmentation toolkit. 2011 IEEE International Symposium on Biomedical Imaging: From Nano to Macro.

[148] Held M, Schmitz MHA, Fischer B, Walter T, Neumann B, Olma MH (2010). CellCognition: time-resolved phenotype annotation in high-throughput live cell imaging. Nat Methods.

[149] Rajaram S, Pavie B, Wu LF, Altschuler SJ (2012). PhenoRipper: software for rapidly profiling microscopy images. Nat Methods.

[150] Shamir L, Orlov N, Eckley DM, Macura T, Johnston J, Goldberg IG (2008). Wndchrm – an open source utility for biological image analysis. Source Code Biol Med.

[151] Schindelin J, Arganda-Carreras I, Frise E, Kaynig V, Longair M, Pietzsch T (2012). Fiji: an open-source platform for biological-image analysis. Nat Methods.

[152] Pau G, Fuchs F, Sklyar O, Boutros M, Huber W (2010). EBImage--an R package for image processing with applications to cellular phenotypes. Bioinformatics..

[153] LeCun Y, Bengio Y, Hinton G (2015). Deep learning. Nature..

[154] Webb S (2018). Deep learning for biology. Nature..

[155] Abadi M, Barham P, Chen J, Chen Z, Davis A, Dean J (2016). TensorFlow: A System for Large-Scale Machine Learning.

[156] van Merriënboer B, Bahdanau D, Dumoulin V, Serdyuk D, Warde-Farley D, Chorowski J, et al. Blocks and Fuel: Frameworks for deep learning. arXiv. 2015:150600619 Available from: http://arxiv.org/abs/1506.00619. [cited 14 Oct 2018].

[157] Collobert R, Kavukcuoglu K (2011). Torch7: a matlab-like environment for machine learning. BigLearn, NIPS Workshop.

[158] Jia Y, Shelhamer E, Donahue J, Karayev S, Long J, Girshick R (2014). Caffe: Convolutional Architecture for Fast Feature Embedding. Proceedings of the ACM International Conference on Multimedia - MM ‘14.

[159] Kim Y. Convolutional Neural Networks for Sentence Classification. arXiv. 2014:14085882 Available from: http://arxiv.org/abs/1408.5882. [cited 14 Oct 2018].

[160] Ronneberger O, Fischer P, Brox T. U-Net: Convolutional Networks for Biomedical Image Segmentation. arXiv. 2015:150504597 Available from: http://arxiv.org/abs/1505.04597. [cited 22 Oct 2018].

[161] Duan L, Qin X, He Y, Sang X, Pan J, Xu T (2018). Segmentation of *Drosophila* Heart in Optical Coherence Microscopy Images Using Convolutional Neural Networks. J Biophotonics.

[162] Liang P, Chen J, Brodskiy PA, Wu Q, Zhang Y, Zhang Y (2018). A new registration approach for dynamic analysis of calcium signals in organs. 2018 IEEE 15th International Symposium on Biomedical Imaging (ISBI 2018).

[163] Li H, Fan Y. Non-rigid image registration using fully convolutional networks with deep self-supervision. 2017;arXiv preprint arXiv:1709.00799.

[164] Pan SJ, Yang Q (2010). A survey on transfer learning. IEEE Trans Knowl Data Eng.

[165] Medici V, Vonesch SC, Fry SN, Hafen E (2015). The FlyCatwalk: a high-throughput feature-based sorting system for artificial selection in Drosophila. G3: genes, genomes. Genetics..

[166] Alisch T, Crall JD, Kao AB, Zucker D, de Bivort BL, Marder E, Calabrese RL, Rankin CH, Gilestro GF (2018). MAPLE (modular automated platform for large-scale experiments), a robot for integrated organism-handling and phenotyping. eLife.

[167] Kennedy BF, Kennedy KM, Sampson DD (2014). A review of optical coherence Elastography: fundamentals, techniques and prospects. IEEE J Sel Top Quantum Electron.

[168] Chen L-C, Zhu Y, Papandreou G, Schroff F, Adam H, Ferrari V, Hebert M, Sminchisescu C, Weiss Y (2018). Encoder-Decoder with Atrous Separable Convolution for Semantic Image Segmentation. Computer Vision – ECCV 2018.

[169] He K, Gkioxari G, Dollár P, Girshick R (2017). Mask R-CNN.

[170] Zhang H, Dana K, Shi J, Zhang Z, Wang X, Tyagi A (2018). Context Encoding for Semantic Segmentation. 2018 IEEE/CVF Conference on Computer Vision and Pattern Recognition.

[171] Mackay DJC (1995). Probable networks and plausible predictions — a review of practical Bayesian methods for supervised neural networks. Netw Comput Neural Syst.

[172] Shen D, Wu G, Suk H-I (2017). Deep learning in medical image analysis. Annu Rev Biomed Eng.

[173] Zhang K, Zuo W, Chen Y, Meng D, Zhang L (2017). Beyond a Gaussian Denoiser: residual learning of deep CNN for image Denoising. IEEE Trans Image Process.

[174] Zhou B, Khosla A, Lapedriza A, Oliva A, Torralba A (2016). Learning Deep Features for Discriminative Localization. 2016 IEEE Conference on Computer Vision and Pattern Recognition (CVPR).

[175] Brodland GW (2015). How computational models can help unlock biological systems. Semin Cell Dev Biol.

[176] Reeves GT, Muratov CB, Schüpbach T, Shvartsman SY (2006). Quantitative models of developmental pattern formation. Dev Cell.

[177] Shvartsman SY, Muratov CB, Lauffenburger DA (2002). Modeling and computational analysis of EGF receptor-mediated cell communication in Drosophila oogenesis. Development..

[178] Lembong J, Yakoby N, Shvartsman SY (2009). Pattern formation by dynamically interacting network motifs. PNAS..

[179] Zartman JJ, Cheung LS, Niepielko MG, Bonini C, Haley B, Yakoby N (2011). Pattern formation by a moving morphogen source. Phys Biol.

[180] Umulis DM, Othmer HG (2015). The role of mathematical models in understanding pattern formation in developmental biology. Bull Math Biol.

[181] Gou J, Lin L, Othmer HG (2018). A model for the hippo pathway in the Drosophila wing disc. Biophys J.

[182] Zartman JJ, Shvartsman SY (2010). Unit operations of tissue development: epithelial folding. Annu Rev Chem Biomol Eng.

[183] Murisic N, Hakim V, Kevrekidis IG, Shvartsman SY, Audoly B (2015). From discrete to continuum models of three-dimensional deformations in epithelial sheets. Biophys J.

[184] Blanchard GB, Fletcher AG, Schumacher LJ (2018). The devil is in the mesoscale: mechanical and behavioural heterogeneity in collective cell movement.

[185] Davidson L, von Dassow M, Zhou J (2009). Multi-scale mechanics from molecules to morphogenesis. Int J Biochem Cell Biol.

[186] Green S, Batterman R (2017). Biology meets physics: reductionism and multi-scale modeling of morphogenesis. Stud Hist Philos Biol Biomed Sci.

[187] Clément R, Dehapiot B, Collinet C, Lecuit T, Lenne P-F (2017). Viscoelastic Dissipation Stabilizes Cell Shape Changes during Tissue Morphogenesis. Curr Biol.

[188] Pajic-Lijakovic I, Milivojevic M (2017). Viscoelasticity of multicellular surfaces. J Biomech.

[189] Pajic-Lijakovic I, Milivojevic M. Long-time viscoelasticity of multicellular surfaces caused by collective cell migration-multi-scale modeling considerations. Semin Cell Dev Biol. 2018. In press. 10.1016/j.semcdb.2018.08.002 30086376

[190] Wyatt T, Baum B, Charras G (2016). A question of time: tissue adaptation to mechanical forces. Curr Opin Cell Biol.

[191] Veldhuis JH, Mashburn D, Hutson MS, Brodland GW (2015). Practical aspects of the cellular force inference toolkit (CellFIT). Methods Cell Biol.

[192] Sugimura K, Lenne P-F, Graner F (2016). Measuring forces and stresses in situ in living tissues. Development..

[193] Veldhuis JH, Ahmad E, Jean-Léon M, Takashi H, Simon C, Wayne BG (2017). Inferring cellular forces from image stacks Philosophical Transactions of the Royal Society B. Biol Sci.

[194] Yevick HG, Martin AC (2018). Quantitative analysis of cell shape and the cytoskeleton in developmental biology. Wiley Interdiscip Rev Dev Biol.

[195] Angelikopoulos P, Papadimitriou C, Koumoutsakos P (2012). Bayesian uncertainty quantification and propagation in molecular dynamics simulations: a high performance computing framework. J Chem Phys.

[196] Levario TJ, Lim B, Shvartsman SY, Lu H (2016). Microfluidics for high-throughput quantitative studies of early development. Annu Rev Biomed Eng.

[197] Kursawe J, Brodskiy PA, Zartman JJ, Baker RE, Fletcher AG (2015). Capabilities and limitations of tissue size control through passive mechanical forces. PLoS Comput Biol.

[198] Meijering E (2012). Cell segmentation: 50 years down the road [life sciences]. IEEE Signal Process Mag.

[199] Dobens AC, Dobens LL (2013). FijiWings: An Open Source Toolkit for Semiautomated Morphometric Analysis of Insect Wings. G3 (Bethesda).

[200] Van Valen DA, Kudo T, Lane KM, Macklin DN, Quach NT, DeFelice MM (2016). Deep Learning Automates the Quantitative Analysis of Individual Cells in Live-Cell Imaging Experiments. PLoS Comput Biol.

